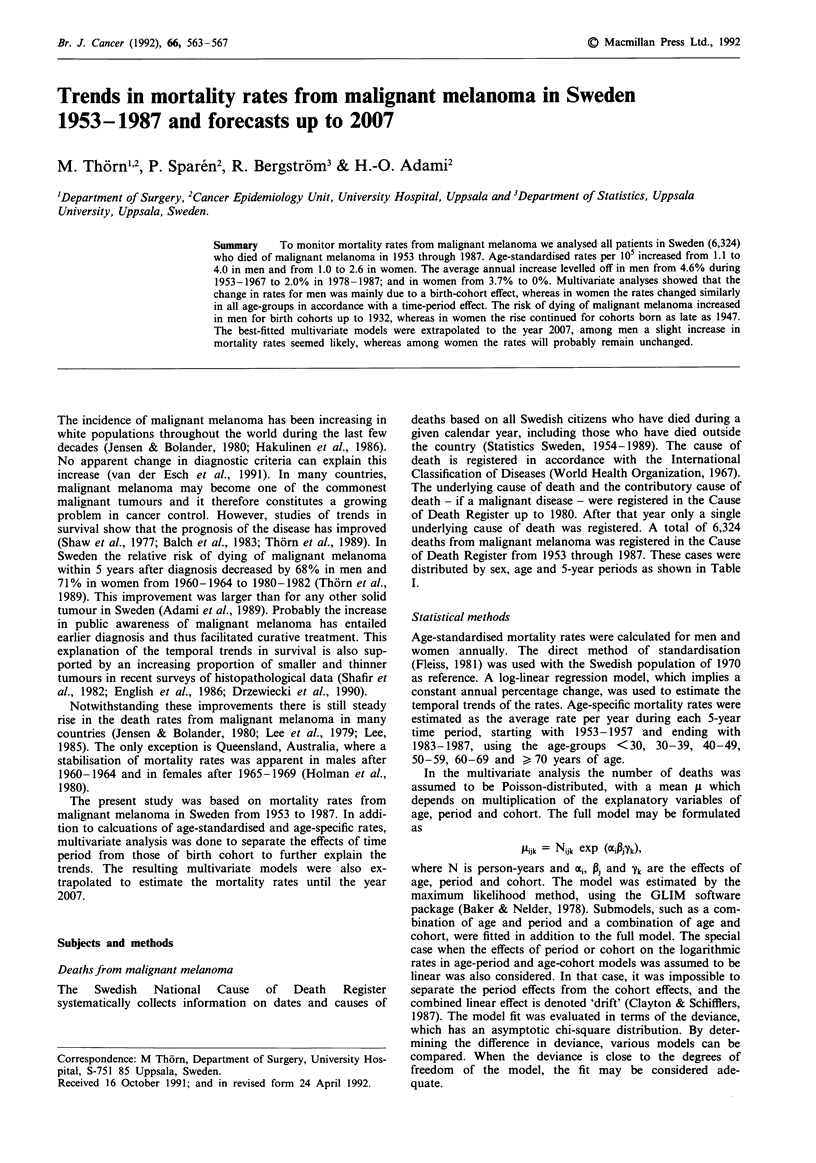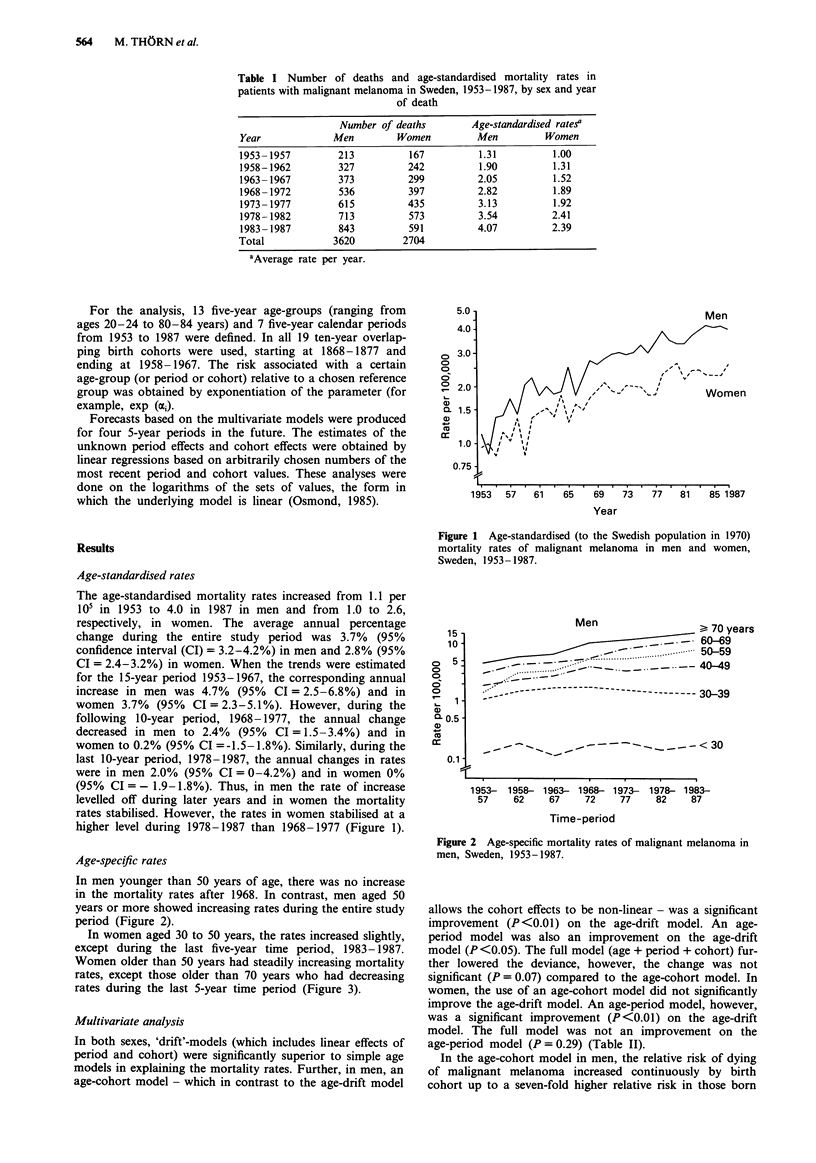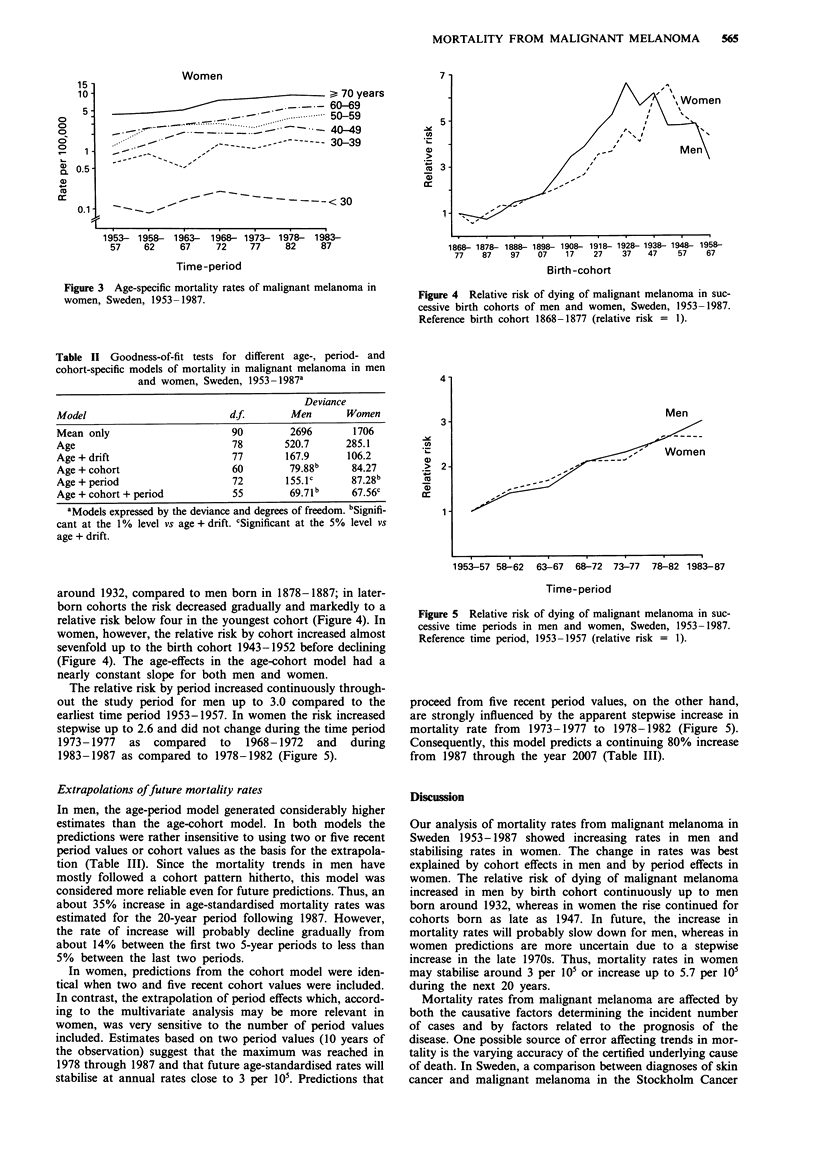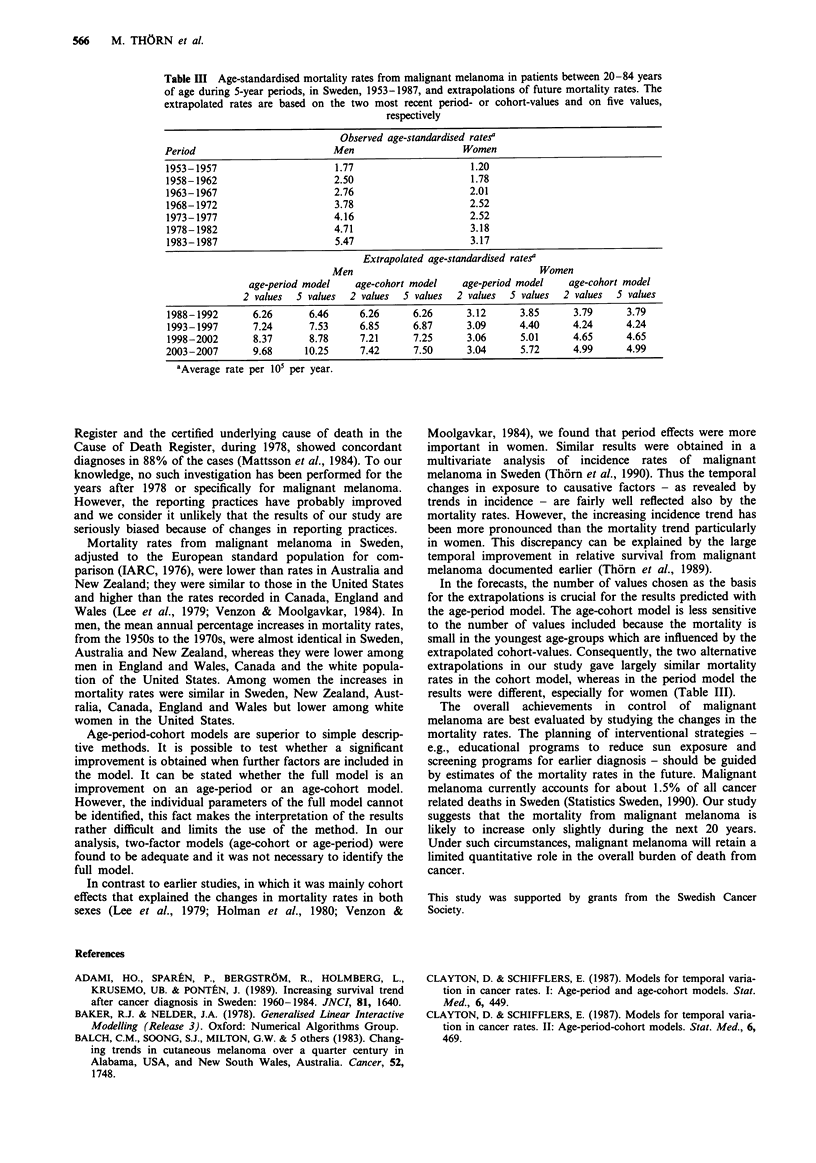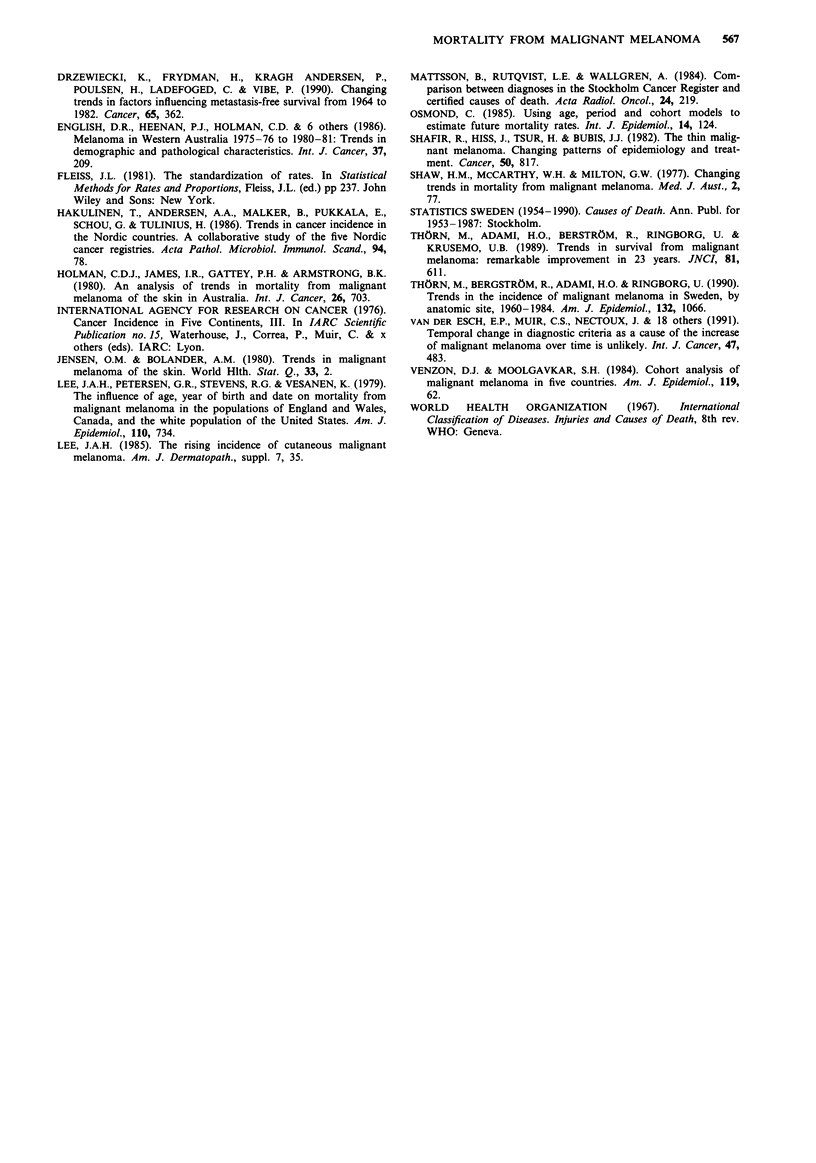# Trends in mortality rates from malignant melanoma in Sweden 1953-1987 and forecasts up to 2007.

**DOI:** 10.1038/bjc.1992.315

**Published:** 1992-09

**Authors:** M. Thörn, P. Sparén, R. Bergström, H. O. Adami

**Affiliations:** Department of Surgery, University Hospital, Uppsala, Sweden.

## Abstract

To monitor mortality rates from malignant melanoma we analysed all patients in Sweden (6,324) who died of malignant melanoma in 1953 through 1987. Age-standardised rates per 10(5) increased from 1.1 to 4.0 in men and from 1.0 to 2.6 in women. The average annual increase levelled off in men from 4.6% during 1953-1967 to 2.0% in 1978-1987; and in women from 3.7% to 0%. Multivariate analyses showed that the change in rates for men was mainly due to a birth-cohort effect, whereas in women the rates changed similarly in all age-groups in accordance with a time-period effect. The risk of dying of malignant melanoma increased in men for birth cohorts up to 1932, whereas in women the rise continued for cohorts born as late as 1947. The best-fitted multivariate models were extrapolated to the year 2007, among men a slight increase in mortality rates seemed likely, whereas among women the rates will probably remain unchanged.


Br. J. Cancer (1992), 66, 563–567 © Macmillan Press Ltd., 1992

### Trends in mortality rates from malignant melanoma in Sweden 1953–1987 and forecasts up to 2007

M. Thörn[1,2], P. Sparén[2], R. Bergström[3] & H.-O. Adami[2]

[1]*Department of Surgery,* [2]*Cancer Epidemiology Unit, University Hospital, Uppsala and* [3]*Department of Statistics, Uppsala University, Uppsala, Sweden.*

**Summary** To monitor mortality rates from malignant melanoma we analysed all patients in Sweden (6,324) who died of malignant melanoma in 1953 through 1987. Age-standardised rates per $10^5$ increased from 1.1 to 4.0 in men and from 1.0 to 2.6 in women. The average annual increase levelled off in men from 4.6% during 1953–1967 to 2.0% in 1978–1987; and in women from 3.7% to 0%. Multivariate analyses showed that the change in rates for men was mainly due to a birth-cohort effect, whereas in women the rates changed similarly in all age-groups in accordance with a time-period effect. The risk of dying of malignant melanoma increased in men for birth cohorts up to 1932, whereas in women the rise continued for cohorts born as late as 1947. The best-fitted multivariate models were extrapolated to the year 2007, among men a slight increase in mortality rates seemed likely, whereas among women the rates will probably remain unchanged.

The incidence of malignant melanoma has been increasing in white populations throughout the world during the last few decades (Jensen & Bolander, 1980; Hakulinen *et al.*, 1986). No apparent change in diagnostic criteria can explain this increase (van der Esch *et al.*, 1991). In many countries, malignant melanoma may become one of the commonest malignant tumours and it therefore constitutes a growing problem in cancer control. However, studies of trends in survival show that the prognosis of the disease has improved (Shaw *et al.*, 1977; Balch *et al.*, 1983; Thörn *et al.*, 1989). In Sweden the relative risk of dying of malignant melanoma within 5 years after diagnosis decreased by 68% in men and 71% in women from 1960–1964 to 1980–1982 (Thörn *et al.*, 1989). This improvement was larger than for any other solid tumour in Sweden (Adami *et al.*, 1989). Probably the increase in public awareness of malignant melanoma has entailed earlier diagnosis and thus facilitated curative treatment. This explanation of the temporal trends in survival is also supported by an increasing proportion of smaller and thinner tumours in recent surveys of histopathological data (Shafir *et al.*, 1982; English *et al.*, 1986; Drzewiecki *et al.*, 1990).

Notwithstanding these improvements there is still steady rise in the death rates from malignant melanoma in many countries (Jensen & Bolander, 1980; Lee *et al.*, 1979; Lee, 1985). The only exception is Queensland, Australia, where a stabilisation of mortality rates was apparent in males after 1960–1964 and in females after 1965–1969 (Holman *et al.*, 1980).

The present study was based on mortality rates from malignant melanoma in Sweden from 1953 to 1987. In addition to calcuations of age-standardised and age-specific rates, multivariate analysis was done to separate the effects of time period from those of birth cohort to further explain the trends. The resulting multivariate models were also extrapolated to estimate the mortality rates until the year 2007.

### Subjects and methods

### *Deaths from malignant melanoma*

The Swedish National Cause of Death Register systematically collects information on dates and causes of deaths based on all Swedish citizens who have died during a given calendar year, including those who have died outside the country (Statistics Sweden, 1954–1989). The cause of death is registered in accordance with the International Classification of Diseases (World Health Organization, 1967). The underlying cause of death and the contributory cause of death – if a malignant disease – were registered in the Cause of Death Register up to 1980. After that year only a single underlying cause of death was registered. A total of 6,324 deaths from malignant melanoma was registered in the Cause of Death Register from 1953 through 1987. These cases were distributed by sex, age and 5-year periods as shown in Table I.

### *Statistical methods*

Age-standardised mortality rates were calculated for men and women annually. The direct method of standardisation (Fleiss, 1981) was used with the Swedish population of 1970 as reference. A log-linear regression model, which implies a constant annual percentage change, was used to estimate the temporal trends of the rates. Age-specific mortality rates were estimated as the average rate per year during each 5-year time period, starting with 1953–1957 and ending with 1983–1987, using the age-groups $<30$, 30–39, 40–49, 50–59, 60–69 and $\geqslant 70$ years of age.

In the multivariate analysis the number of deaths was assumed to be Poisson-distributed, with a mean $\mu$ which depends on multiplication of the explanatory variables of age, period and cohort. The full model may be formulated as

$$\mu_{ijk} = N_{ijk} \exp(\alpha_i \beta_j \gamma_k),$$

where N is person-years and $\alpha_i$, $\beta_j$ and $\gamma_k$ are the effects of age, period and cohort. The model was estimated by the maximum likelihood method, using the GLIM software package (Baker & Nelder, 1978). Submodels, such as a combination of age and period and a combination of age and cohort, were fitted in addition to the full model. The special case when the effects of period or cohort on the logarithmic rates in age-period and age-cohort models was assumed to be linear was also considered. In that case, it was impossible to separate the period effects from the cohort effects, and the combined linear effect is denoted 'drift' (Clayton & Schifflers, 1987). The model fit was evaluated in terms of the deviance, which has an asymptotic chi-square distribution. By determining the difference in deviance, various models can be compared. When the deviance is close to the degrees of freedom of the model, the fit may be considered adequate.

Correspondence: M Thörn, Department of Surgery, University Hospital, S-751 85 Uppsala, Sweden.
Received 16 October 1991; and in revised form 24 April 1992.

**Table I** Number of deaths and age-standardised mortality rates in patients with malignant melanoma in Sweden, 1953–1987, by sex and year of death

| Year | *Number of deaths* Men | Women | *Age-standardised rates*[a] Men | Women |
|---|---|---|---|---|
| 1953–1957 | 213 | 167 | 1.31 | 1.00 |
| 1958–1962 | 327 | 242 | 1.90 | 1.31 |
| 1963–1967 | 373 | 299 | 2.05 | 1.52 |
| 1968–1972 | 536 | 397 | 2.82 | 1.89 |
| 1973–1977 | 615 | 435 | 3.13 | 1.92 |
| 1978–1982 | 713 | 573 | 3.54 | 2.41 |
| 1983–1987 | 843 | 591 | 4.07 | 2.39 |
| Total | 3620 | 2704 | | |

[a]Average rate per year.

For the analysis, 13 five-year age-groups (ranging from ages 20–24 to 80–84 years) and 7 five-year calendar periods from 1953 to 1987 were defined. In all 19 ten-year overlapping birth cohorts were used, starting at 1868–1877 and ending at 1958–1967. The risk associated with a certain age-group (or period or cohort) relative to a chosen reference group was obtained by exponentiation of the parameter (for example, exp ($\alpha_i$).

Forecasts based on the multivariate models were produced for four 5-year periods in the future. The estimates of the unknown period effects and cohort effects were obtained by linear regressions based on arbitrarily chosen numbers of the most recent period and cohort values. These analyses were done on the logarithms of the sets of values, the form in which the underlying model is linear (Osmond, 1985).

**Figure 1** Age-standardised (to the Swedish population in 1970) mortality rates of malignant melanoma in men and women, Sweden, 1953–1987.

### Results

### *Age-standardised rates*

The age-standardised mortality rates increased from 1.1 per $10^5$ in 1953 to 4.0 in 1987 in men and from 1.0 to 2.6, respectively, in women. The average annual percentage change during the entire study period was 3.7% (95% confidence interval (CI) = 3.2–4.2%) in men and 2.8% (95% CI = 2.4–3.2%) in women. When the trends were estimated for the 15-year period 1953–1967, the corresponding annual increase in men was 4.7% (95% CI = 2.5–6.8%) and in women 3.7% (95% CI = 2.3–5.1%). However, during the following 10-year period, 1968–1977, the annual change decreased in men to 2.4% (95% CI = 1.5–3.4%) and in women to 0.2% (95% CI = -1.5–1.8%). Similarly, during the last 10-year period, 1978–1987, the annual changes in rates were in men 2.0% (95% CI = 0–4.2%) and in women 0% (95% CI = – 1.9–1.8%). Thus, in men the rate of increase levelled off during later years and in women the mortality rates stabilised. However, the rates in women stabilised at a higher level during 1978–1987 than 1968–1977 (Figure 1).

**Figure 2** Age-specific mortality rates of malignant melanoma in men, Sweden, 1953–1987.

### *Age-specific rates*

In men younger than 50 years of age, there was no increase in the mortality rates after 1968. In contrast, men aged 50 years or more showed increasing rates during the entire study period (Figure 2).

In women aged 30 to 50 years, the rates increased slightly, except during the last five-year time period, 1983–1987. Women older than 50 years had steadily increasing mortality rates, except those older than 70 years who had decreasing rates during the last 5-year time period (Figure 3).

### *Multivariate analysis*

In both sexes, 'drift'-models (which includes linear effects of period and cohort) were significantly superior to simple age models in explaining the mortality rates. Further, in men, an age-cohort model – which in contrast to the age-drift model allows the cohort effects to be non-linear – was a significant improvement ($P < 0.01$) on the age-drift model. An age-period model was also an improvement on the age-drift model ($P < 0.05$). The full model (age + period + cohort) further lowered the deviance, however, the change was not significant ($P = 0.07$) compared to the age-cohort model. In women, the use of an age-cohort model did not significantly improve the age-drift model. An age-period model, however, was a significant improvement ($P < 0.01$) on the age-drift model. The full model was not an improvement on the age-period model ($P = 0.29$) (Table II).

In the age-cohort model in men, the relative risk of dying of malignant melanoma increased continuously by birth cohort up to a seven-fold higher relative risk in those born

**Figure 3** Age-specific mortality rates of malignant melanoma in women, Sweden, 1953–1987.

**Figure 4** Relative risk of dying of malignant melanoma in successive birth cohorts of men and women, Sweden, 1953–1987. Reference birth cohort 1868–1877 (relative risk = 1).

**Table II** Goodness-of-fit tests for different age-, period- and cohort-specific models of mortality in malignant melanoma in men and women, Sweden, 1953–1987[a]

| Model | d.f. | Deviance Men | Women |
|---|---|---|---|
| Mean only | 90 | 2696 | 1706 |
| Age | 78 | 520.7 | 285.1 |
| Age + drift | 77 | 167.9 | 106.2 |
| Age + cohort | 60 | 79.88[b] | 84.27 |
| Age + period | 72 | 155.1[c] | 87.28[b] |
| Age + cohort + period | 55 | 69.71[b] | 67.56[c] |

[a]Models expressed by the deviance and degrees of freedom. [b]Significant at the 1% level *vs* age + drift. [c]Significant at the 5% level *vs* age + drift.

**Figure 5** Relative risk of dying of malignant melanoma in successive time periods in men and women, Sweden, 1953–1987. Reference time period, 1953–1957 (relative risk = 1).

around 1932, compared to men born in 1878–1887; in later-born cohorts the risk decreased gradually and markedly to a relative risk below four in the youngest cohort (Figure 4). In women, however, the relative risk by cohort increased almost sevenfold up to the birth cohort 1943–1952 before declining (Figure 4). The age-effects in the age-cohort model had a nearly constant slope for both men and women.

The relative risk by period increased continuously throughout the study period for men up to 3.0 compared to the earliest time period 1953–1957. In women the risk increased stepwise up to 2.6 and did not change during the time period 1973–1977 as compared to 1968–1972 and during 1983–1987 as compared to 1978–1982 (Figure 5).

### *Extrapolations of future mortality rates*

In men, the age-period model generated considerably higher estimates than the age-cohort model. In both models the predictions were rather insensitive to using two or five recent period values or cohort values as the basis for the extrapolation (Table III). Since the mortality trends in men have mostly followed a cohort pattern hitherto, this model was considered more reliable even for future predictions. Thus, an about 35% increase in age-standardised mortality rates was estimated for the 20-year period following 1987. However, the rate of increase will probably decline gradually from about 14% between the first two 5-year periods to less than 5% between the last two periods.

In women, predictions from the cohort model were identical when two and five recent cohort values were included. In contrast, the extrapolation of period effects which, according to the multivariate analysis may be more relevant in women, was very sensitive to the number of period values included. Estimates based on two period values (10 years of the observation) suggest that the maximum was reached in 1978 through 1987 and that future age-standardised rates will stabilise at annual rates close to 3 per $10^5$. Predictions that proceed from five recent period values, on the other hand, are strongly influenced by the apparent stepwise increase in mortality rate from 1973–1977 to 1978–1982 (Figure 5). Consequently, this model predicts a continuing 80% increase from 1987 through the year 2007 (Table III).

### Discussion

Our analysis of mortality rates from malignant melanoma in Sweden 1953–1987 showed increasing rates in men and stabilising rates in women. The change in rates was best explained by cohort effects in men and by period effects in women. The relative risk of dying of malignant melanoma increased in men by birth cohort continuously up to men born around 1932, whereas in women the rise continued for cohorts born as late as 1947. In future, the increase in mortality rates will probably slow down for men, whereas in women predictions are more uncertain due to a stepwise increase in the late 1970s. Thus, mortality rates in women may stabilise around 3 per $10^5$ or increase up to 5.7 per $10^5$ during the next 20 years.

Mortality rates from malignant melanoma are affected by both the causative factors determining the incident number of cases and by factors related to the prognosis of the disease. One possible source of error affecting trends in mortality is the varying accuracy of the certified underlying cause of death. In Sweden, a comparison between diagnoses of skin cancer and malignant melanoma in the Stockholm Cancer

**Table III** Age-standardised mortality rates from malignant melanoma in patients between 20–84 years of age during 5-year periods, in Sweden, 1953–1987, and extrapolations of future mortality rates. The extrapolated rates are based on the two most recent period- or cohort-values and on five values, respectively

| *Period* | *Observed age-standardised rates*[a] *Men* | *Women* |
|---|---|---|
| 1953–1957 | 1.77 | 1.20 |
| 1958–1962 | 2.50 | 1.78 |
| 1963–1967 | 2.76 | 2.01 |
| 1968–1972 | 3.78 | 2.52 |
| 1973–1977 | 4.16 | 2.52 |
| 1978–1982 | 4.71 | 3.18 |
| 1983–1987 | 5.47 | 3.17 |

| | *Extrapolated age-standardised rates*[a] *Men* | | | | *Women* | | | |
|---|---|---|---|---|---|---|---|---|
| | *age-period model* | | *age-cohort model* | | *age-period model* | | *age-cohort model* | |
| | *2 values* | *5 values* | *2 values* | *5 values* | *2 values* | *5 values* | *2 values* | *5 values* |
| 1988–1992 | 6.26 | 6.46 | 6.26 | 6.26 | 3.12 | 3.85 | 3.79 | 3.79 |
| 1993–1997 | 7.24 | 7.53 | 6.85 | 6.87 | 3.09 | 4.40 | 4.24 | 4.24 |
| 1998–2002 | 8.37 | 8.78 | 7.21 | 7.25 | 3.06 | 5.01 | 4.65 | 4.65 |
| 2003–2007 | 9.68 | 10.25 | 7.42 | 7.50 | 3.04 | 5.72 | 4.99 | 4.99 |

[a]Average rate per $10^5$ per year.

Register and the certified underlying cause of death in the Cause of Death Register, during 1978, showed concordant diagnoses in 88% of the cases (Mattsson *et al.*, 1984). To our knowledge, no such investigation has been performed for the years after 1978 or specifically for malignant melanoma. However, the reporting practices have probably improved and we consider it unlikely that the results of our study are seriously biased because of changes in reporting practices.

Mortality rates from malignant melanoma in Sweden, adjusted to the European standard population for comparison (IARC, 1976), were lower than rates in Australia and New Zealand; they were similar to those in the United States and higher than the rates recorded in Canada, England and Wales (Lee *et al.*, 1979; Venzon & Moolgavkar, 1984). In men, the mean annual percentage increases in mortality rates, from the 1950s to the 1970s, were almost identical in Sweden, Australia and New Zealand, whereas they were lower among men in England and Wales, Canada and the white population of the United States. Among women the increases in mortality rates were similar in Sweden, New Zealand, Australia, Canada, England and Wales but lower among white women in the United States.

Age-period-cohort models are superior to simple descriptive methods. It is possible to test whether a significant improvement is obtained when further factors are included in the model. It can be stated whether the full model is an improvement on an age-period or an age-cohort model. However, the individual parameters of the full model cannot be identified, this fact makes the interpretation of the results rather difficult and limits the use of the method. In our analysis, two-factor models (age-cohort or age-period) were found to be adequate and it was not necessary to identify the full model.

In contrast to earlier studies, in which it was mainly cohort effects that explained the changes in mortality rates in both sexes (Lee *et al.*, 1979; Holman *et al.*, 1980; Venzon & Moolgavkar, 1984), we found that period effects were more important in women. Similar results were obtained in a multivariate analysis of incidence rates of malignant melanoma in Sweden (Thörn *et al.*, 1990). Thus the temporal changes in exposure to causative factors – as revealed by trends in incidence – are fairly well reflected also by the mortality rates. However, the increasing incidence trend has been more pronounced than the mortality trend particularly in women. This discrepancy can be explained by the large temporal improvement in relative survival from malignant melanoma documented earlier (Thörn *et al.*, 1989).

In the forecasts, the number of values chosen as the basis for the extrapolations is crucial for the results predicted with the age-period model. The age-cohort model is less sensitive to the number of values included because the mortality is small in the youngest age-groups which are influenced by the extrapolated cohort-values. Consequently, the two alternative extrapolations in our study gave largely similar mortality rates in the cohort model, whereas in the period model the results were different, especially for women (Table III).

The overall achievements in control of malignant melanoma are best evaluated by studying the changes in the mortality rates. The planning of interventional strategies – e.g., educational programs to reduce sun exposure and screening programs for earlier diagnosis – should be guided by estimates of the mortality rates in the future. Malignant melanoma currently accounts for about 1.5% of all cancer related deaths in Sweden (Statistics Sweden, 1990). Our study suggests that the mortality from malignant melanoma is likely to increase only slightly during the next 20 years. Under such circumstances, malignant melanoma will retain a limited quantitative role in the overall burden of death from cancer.

This study was supported by grants from the Swedish Cancer Society.

**References**

ADAMI, HO., SPARÉN, P., BERGSTRÖM, R., HOLMBERG, L., KRUSEMO, UB. & PONTÉN, J. (1989). Increasing survival trend after cancer diagnosis in Sweden: 1960–1984. *JNCI*, **81**, 1640.

BAKER, R.J. & NELDER, J.A. (1978). *Generalised Linear Interactive Modelling (Release 3)*. Oxford: Numerical Algorithms Group.

BALCH, C.M., SOONG, S.J., MILTON, G.W. & 5 others (1983). Changing trends in cutaneous melanoma over a quarter century in Alabama, USA, and New South Wales, Australia. *Cancer*, **52**, 1748.

CLAYTON, D. & SCHIFFLERS, E. (1987). Models for temporal variation in cancer rates. I: Age-period and age-cohort models. *Stat. Med.*, **6**, 449.

CLAYTON, D. & SCHIFFLERS, E. (1987). Models for temporal variation in cancer rates. II: Age-period-cohort models. *Stat. Med.*, **6**, 469.

DRZEWIECKI, K., FRYDMAN, H., KRAGH ANDERSEN, P., POULSEN, H., LADEFOGED, C. & VIBE, P. (1990). Changing trends in factors influencing metastasis-free survival from 1964 to 1982. *Cancer*, **65**, 362.

ENGLISH, D.R., HEENAN, P.J., HOLMAN, C.D. & 6 others (1986). Melanoma in Western Australia 1975–76 to 1980–81: Trends in demographic and pathological characteristics. *Int. J. Cancer*, **37**, 209.

FLEISS, J.L. (1981). The standardization of rates. In *Statistical Methods for Rates and Proportions*, Fleiss, J.L. (ed.) pp 237. John Wiley and Sons: New York.

HAKULINEN, T., ANDERSEN, A.A., MALKER, B., PUKKALA, E., SCHOU, G. & TULINIUS, H. (1986). Trends in cancer incidence in the Nordic countries. A collaborative study of the five Nordic cancer registries. *Acta Pathol. Microbiol. Immunol. Scand.*, **94**, 78.

HOLMAN, C.D.J., JAMES, I.R., GATTEY, P.H. & ARMSTRONG, B.K. (1980). An analysis of trends in mortality from malignant melanoma of the skin in Australia. *Int. J. Cancer*, **26**, 703.

INTERNATIONAL AGENCY FOR RESEARCH ON CANCER (1976). Cancer Incidence in Five Continents, III. In *IARC Scientific Publication no. 15*, Waterhouse, J., Correa, P., Muir, C. & x others (eds). IARC: Lyon.

JENSEN, O.M. & BOLANDER, A.M. (1980). Trends in malignant melanoma of the skin. World Hlth. *Stat. Q.*, **33**, 2.

LEE, J.A.H., PETERSEN, G.R., STEVENS, R.G. & VESANEN, K. (1979). The influence of age, year of birth and date on mortality from malignant melanoma in the populations of England and Wales, Canada, and the white population of the United States. *Am. J. Epidemiol.*, **110**, 734.

LEE, J.A.H. (1985). The rising incidence of cutaneous malignant melanoma. *Am. J. Dermatopath.*, suppl. 7, 35.

MATTSSON, B., RUTQVIST, L.E. & WALLGREN, A. (1984). Comparison between diagnoses in the Stockholm Cancer Register and certified causes of death. *Acta Radiol. Oncol.*, **24**, 219.

OSMOND, C. (1985). Using age, period and cohort models to estimate future mortality rates. *Int. J. Epidemiol.*, **14**, 124.

SHAFIR, R., HISS, J., TSUR, H. & BUBIS, J.J. (1982). The thin malignant melanoma. Changing patterns of epidemiology and treatment. *Cancer*, **50**, 817.

SHAW, H.M., MCCARTHY, W.H. & MILTON, G.W. (1977). Changing trends in mortality from malignant melanoma. *Med. J. Aust.*, **2**, 77.

STATISTICS SWEDEN (1954–1990). *Causes of Death*. Ann. Publ. for 1953–1987: Stockholm.

THÖRN, M., ADAMI, H.O., BERSTRÖM, R., RINGBORG, U. & KRUSEMO, U.B. (1989). Trends in survival from malignant melanoma: remarkable improvement in 23 years. *JNCI*, **81**, 611.

THÖRN, M., BERGSTRÖM, R., ADAMI, H.O. & RINGBORG, U. (1990). Trends in the incidence of malignant melanoma in Sweden, by anatomic site, 1960–1984. *Am. J. Epidemiol.*, **132**, 1066.

VAN DER ESCH, E.P., MUIR, C.S., NECTOUX, J. & 18 others (1991). Temporal change in diagnostic criteria as a cause of the increase of malignant melanoma over time is unlikely. *Int. J. Cancer*, **47**, 483.

VENZON, D.J. & MOOLGAVKAR, S.H. (1984). Cohort analysis of malignant melanoma in five countries. *Am. J. Epidemiol.*, **119**, 62.

WORLD HEALTH ORGANIZATION (1967). *International Classification of Diseases. Injuries and Causes of Death*, 8th rev. WHO: Geneva.